# 

**Published:** 1919-11-08

**Authors:** 


**The Modern Newspaper of Administrative Medicine and Institutional Life.**

Telegraphic Address: OSPEDALE. RAND, LONDON. Telephone Number: 2734 GERRARD

No. 1744, Vol. LXVII. | Saturday, November 8th, 1919. | PRICE TWOPENCE

